# Different Effects of Transcranial Direct Current Stimulation on Leg Muscle Glucose Uptake Asymmetry in Two Women with Multiple Sclerosis

**DOI:** 10.3390/brainsci10080549

**Published:** 2020-08-13

**Authors:** Alexandra C. Fietsam, Craig D. Workman, Laura L. Boles Ponto, John Kamholz, Thorsten Rudroff

**Affiliations:** 1 Department of Health and Human Physiology, University of Iowa, Iowa City, IA 52242, USA; alexandra-fietsam@uiowa.edu (A.C.F.); craig-workman@uiowa.edu (C.D.W.); 2 Department of Radiology, University of Iowa Hospitals and Clinics, Iowa City, IA 52242, USA; laura-ponto@uiowa.edu; 3 Department of Neurology, University of Iowa Hospitals and Clinics, Iowa City, IA 52242, USA; john-kamholz@uiowa.edu

**Keywords:** tDCS, neuroimaging, positron emission tomography, walking, asymmetries, multiple sclerosis

## Abstract

Asymmetrical lower limb strength is a significant contributor to impaired walking abilities in people with multiple sclerosis (PwMS). Transcranial direct current stimulation (tDCS) may be an effective technique to enhance cortical excitability and increase neural drive to more-affected lower limbs. A sham-controlled, randomized, cross-over design was employed. Two women with MS underwent two 20 min sessions of either 3 mA tDCS or Sham before 20 min of treadmill walking at a self-selected speed. During walking, the participants were injected with the glucose analogue, [^18^F] fluorodeoxyglucose (FDG). Participants were then imaged to examine glucose metabolism and uptake asymmetries in the legs. Standardized uptake values (SUVs) were compared between the legs and asymmetry indices were calculated. Subject 2 was considered physically active (self-reported participating in at least 30 min of moderate-intensity physical activity on at least three days of the week for the last three months), while Subject 1 was physically inactive. In Subject 1, there was a decrease in SUVs at the left knee flexors, left upper leg, left and right plantar flexors, and left and right lower legs and SUVs in the knee extensors and dorsiflexors were considered symmetric after tDCS compared to Sham. Subject 2 showed an increase in SUVs at the left and right upper legs, right plantar flexors, and right lower leg with no muscle group changing asymmetry status. This study demonstrates that tDCS may increase neural drive to leg muscles and decrease glucose uptake during walking in PwMS with low physical activity levels.

## 1. Introduction

With a prevalence of 50–300 cases per 100,000 people, [1] multiple sclerosis (MS) is an inflammatory autoimmune disease of the central nervous system (CNS) [2] characterized by the demyelination and neurodegeneration of CNS axons [3,4]. Consequently, the transmission of information between the CNS and the periphery is often slowed or blocked, leading to various motor and/or sensory symptoms [5]. Impaired walking ability is common in people with MS (PwMS), with reports of over 50% of patients requiring assistance within 18 years of diagnosis [6]. This progressive worsening of independent ambulation has a significant negative impact on daily functional activities and social participation and is perceived by PwMS to be one of the most important factors leading to poor quality of life [7,8]. Importantly, one of the first signs of MS is weakness in one limb, particularly in the lower limbs, which has been found to be a significant contributor to the progressive worsening of walking abilities [9,10]. Furthermore, adequate methods to correct strength asymmetries and improve walking performance in PwMS are underdeveloped. Determining effective treatments is highly significant because muscle function asymmetries and limb loading asymmetries in PwMS occur early in the disease and may worsen over time [11,12,13].

Transcranial direct current stimulation (tDCS) is a non-invasive means of increasing cortical excitability and may be an effective supplementary treatment for PwMS [14]. tDCS uses weak currents applied to the scalp to alter the excitability of underlying cortical neurons [14]. It has a favorable safely profile and only transient adverse side effects (e.g., temporary tingling sensations) [15]. Although tDCS has been shown to increase muscle strength and fatigue resistance in healthy subjects and people with movement disorders [16,17,18,19,20,21,22,23,24], studies examining the effects of tDCS on walking are scarce and inconsistent [25,26,27,28]. The large variability observed in the stimulation parameters (e.g., stimulation site, intensity, and timing) and dependent variables might partly explain these inconclusive findings.

Electromyography (EMG) is a common and convenient method to noninvasively assess muscle activation patterns. However, surface EMG has critical disadvantages such as the limited number of muscles that can be measured simultaneously, the need for a reference contraction for inter-muscular comparisons (i.e., normalization), the inability to measure deep-lying muscles and deeper aspects of superficial muscles, and confounding factors (crosstalk and adipose tissue) [29,30]. Positron emission tomography (PET) imaging of [^18^F] fluorodeoxyglucose (FDG), a glucose analogue, is a feasible approach to simultaneously and objectively evaluate the activity of multiple lower limb muscles after physical activity, and, therefore, circumvent the disadvantages encountered with EMG [31,32]. It is well accepted that glucose is the main energy source for exercising skeletal muscles. Thus, increased glucose uptake (GU) and GU asymmetry between muscles would be associated with a greater metabolic cost of walking, increased energy demands [33,34,35], and early fatigability [10,36] in PwMS. A previous study [37] used positron emission tomography (PET) with FDG as a glucose analog and found that mildly disabled PwMS had greater FDG uptake in the knee and hip flexor muscles than healthy controls after 15 min of treadmill walking at a self-selected speed. Furthermore, FDG uptake was significantly lower in the knee flexors of the weaker compared to the stronger leg, indicating GU asymmetry and that the greater metabolic cost during walking was a compensatory mechanism that may contribute to increased performance fatigability and impaired walking. Taken together, these studies suggested that determining glucose uptake may be an adequate indicator of muscle activation patterns and asymmetries during walking in PwMS.

The purpose of this study was to examine the effects of a single session of tDCS applied over the motor cortex representation for the more-affected leg on glucose uptake asymmetry during treadmill walking in PwMS. We expected decreased asymmetry between the legs and greater muscle activation in the more-affected leg, as indicated by increased glucose uptake, after tDCS in PwMS. Here, we present the effects of tDCS before treadmill walking on glucose uptake in two PwMS.

## 2. Materials and Methods

### 2.1. Participants

Two women with a positive relapsing-remitting MS diagnosis from a movement disorder specialist according to the 2017 revised McDonald’s criteria [38] were recruited from the community. Participants were included if they: (1) were between the ages of 18–70 years old, (2) were moderately disabled (as indicated by a score of 2–6 on the Patient Determined Disease Steps (PDDS) [39]), (3) had a self-reported difference in strength between legs, and (4) were able to walk for 20 min. Exclusion criteria were: (1) relapse within the last 60 days, (2) inability to fast for 6 h, (3) high risk for cardiovascular disease (American College of Sports Medicine (ACSM) risk classification [40]), (4) changes in disease-modifying medications within the last 45 days, (5) concurrent neurological/neuromuscular disease, (6) hospitalization within the last 90 days, (7) diagnosed depression, (8) pregnancy, (9) history of seizures or on medication known to lower seizure threshold, and (10) inability to understand/sign the consent form. The study was conducted in accordance with the Declaration of Helsinki and was approved by the Institutional Review Board at the University of Iowa (IRB ID #201905825). All participants signed informed consent before participating.

### 2.2. Experimental Protocol

**Figure 1:**
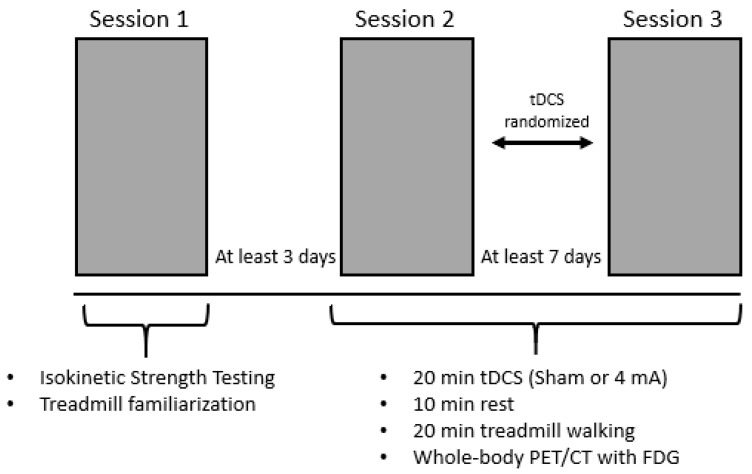
Experimental protocol. Subjects attended three sessions. During Session 1, subjects performed isokinetic strength testing to objectively determine their more-affected leg. During Sessions 2 and 3, participants underwent 20 min of either Sham or 3 mA transcranial direct current stimulation (tDCS) (determined through randomization) over the motor cortex (M1) area corresponding to their more-affected leg, followed by 20 min of treadmill walking after a 10 min rest, then a whole-body positron emission tomography/computed tomography (PET/CT scan with fluorodeoxyglucose (FDG)).

A single-blind, sham-controlled, randomized, cross-over design was employed. Subjects attended three experimental sessions (Figure 1). In the first session, participants gave consent, completed the isokinetic strength testing of the knee extensors and flexors to objectively determine the more-affected leg, and self-selected a comfortable walking speed on the treadmill to be used during sessions 2 and 3. At least three days after the first session, Session 2 was completed. Sessions 2 and 3 were separated by at least seven days to allow tDCS effects to dissipate [41,42]. Prior to sessions 2 and 3, the participants were asked to fast for a minimum of 6 h before FDG administration. At the beginning of sessions 2 and 3, the participants’ height and weight were measured, blood glucose levels were determined, and an IV catheter was inserted for FDG injection. Blood glucose level was required to be equal to or less than 200 mg/dL in order to proceed with FDG administration and PET scanning [43,44]. During sessions 2 and 3, either Sham (0 mA) or tDCS (3 mA) was applied (determined through randomization) to the motor cortex (M1) area corresponding to the more-affected leg for 20 min [45]. The purpose (e.g., enhancing motor learning or improving motor performance) of tDCS may determine stimulation timing. Studies on motor learning often apply tDCS during a task to improve acquisition [46,47]. In contrast, tDCS was applied before the motor performance of a well-learned task (walking) in the current study. It has previously been demonstrated that tDCS has ambiguous effects on cortical excitability during stimulation, but significantly and consistently increased cortical excitability after stimulation [48]. Moreover, when comparing tDCS before and during a 6-min walk test in PwMS, it was found that stimulation before increased gait velocity, whereas stimulation during resulted in a decrease in the distance walked [22]. Therefore, in this study participants underwent 20 min of tDCS then rested for 10 min to allow for peak stimulation effects [20,48] before walking on a treadmill for 20 min. Ratings of Perceived Exhaustion (RPE) were collected at the end of each minute during the walking task. Approximately two minutes into treadmill walking, 10 ± 10% mCi of FDG was administered via IV injection. Immediately after walking was completed, participants were positioned in the PET/computed tomography (CT) scanner and a whole body (top of head to toes) scan was completed to evaluate glucose uptake in the leg muscles.

### 2.3. Isokinetic Strength Testing

Strength testing was completed with an isokinetic dynamometer (HUMAC NORM, CSMi, Stoughton, MA, USA). Before strength testing, participants completed a submaximal warm-up of the knee extensors and flexors consisting of 15 concentric/concentric repetitions at 60°/s. Participants rested for at least 30 s before performing five sets of one repetition maximal effort knee extension and flexion (concentric/concentric, 60°/s) on one leg [49]. After a two-minute rest, participants completed the identical strength testing protocol on the opposite leg. Administrators provided verbal encouragement during the test to promote a maximal effort performance from the participants. The torques produced in each set by each leg were compared to determine the more-affected leg for each participant.

### 2.4. tDCS

tDCS was delivered with a battery-powered stimulator (Soterix Medical Inc., New York, NY, USA) via two carbon electrodes inserted into 5 cm × 7 cm saline soaked sponges. The anode was placed over the motor cortex area corresponding to the more-affected leg for each participant (C3 or C4, using the 10–20 electroencephalography (EEG) placement convention [50]) and the cathode was placed over the supraorbital area on the contralateral side. Similar to previous studies targeting the unilateral M1 leg area [51,52] using this montage, the relatively large anode covered the center of the skull (Cz). Therefore, the anode also covered the leg area, located in the longitudinal fissure [53], corresponding to the more-affected lower limb. The electrode was positioned with a 45° deviation from the sagittal plane to allow for alignment with the motor cortex [53]. During tDCS, the stimulation ramped-up over a 30 s period, remained at 3 mA for 20 min, then ramped-down to 0 mA over a 30 s period. During Sham, the stimulation was ramped-up to 3 mA over a 30 s period then immediately ramped-down to 0 mA over a 30 s period at the beginning and end of the session, but otherwise remained at 0 mA. After tDCS, participants were asked to describe the sensations (e.g., itching, burning, tingling) they experienced during stimulation and the severity of those sensation on a 10-point (1 = “barely perceptible” and 10 = “most I could possibly stand”) Likert-type scale. Participants were also asked to indicate which stimulation intensity (0 mA (Sham) or 3 mA (tDCS)) they thought they had received. Their responses were recorded, but participant blinding was not broken until the final session was completed.

### 2.5. PET/CT Imaging

Two minutes into the walking test on the treadmill, a Certified Nuclear Medicine Technologist injected ~10 mCi of FDG followed by a 20 mL saline flush into an indwelling antecubital catheter. Upon completion of the 20 min walking test, the participants were immediately guided into the scanner and the acquisition and processing of the PET/CT images was performed following a standard protocol used in the University of Iowa Hospital and Clinics.

The PET/CT scans were performed with a GE Discovery MI Time of Flight PET/CT scanner with SiPM array detector technology (GE Healthcare, Waukesha, WI, USA). The PET scans were preceded by CT scans for attenuation correction and for anatomical reference. Both the PET and CT scans were performed with the participants on the same scanning table and in the same position. The body of the subject was secured to maintain co-registration. The whole body was scanned with a scan duration of 2–5 min per bed position depending on the body mass index (BMI) of the subject and 5 min per bed position for the legs. The data sets were reconstructed using an OSEM 3D reconstruction with 16 subsets and three iterations with a Gaussian smoothing of 5 mm. All data sets were corrected for scatter, dead-time, and random coincidence.

### 2.6. Data Analysis

**Figure 2:**
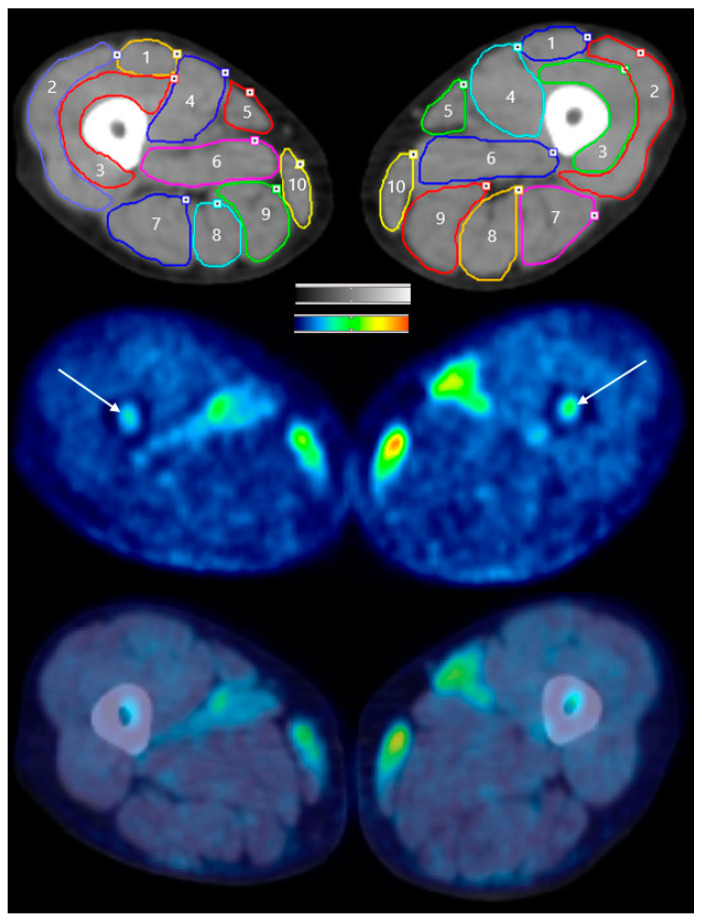
Cross-sectional images at the mid-thigh level of Subject 2 in the tDCS condition. The top image is a computed tomography (CT) scan with regions of interest (ROIs) circled to indicate the location of the skeletal muscles. 1 = Rectus Femoris, 2 = Vastus Lateralis, 3 = Vastus Intermedius, 4 = Vastus Medialis, 5 = Sartorius, 6 = Adductor Magnus (not included in the analysis), 7 = Biceps Femoris, 8 = Semitendinosus, 9 = Semimembranosus, 10 = Gracilis. The ROIs drawn on the CT image were transferred to the positron emission tomography (PET) image (middle image). The white arrows indicate bone marrow metabolism. The bottom image is of the co-registered CT and PET images. Red denotes the greatest signal intensity, followed by yellow, green, and blue.

Twenty regions of interest (ROIs) were drawn on the CT scan from each experimental session to locate the skeletal muscles of the lower limbs. In the upper leg, the knee extensors (rectus femoris, vastus medialis, vastus intermedius, and vastus lateralis) and knee flexors (long and short head of the biceps femoris, semimembranosus, semitendinosus, sartorius, and gracilis) were identified. In the lower leg, the plantar flexors (gastrocnemius, soleus, peroneus longus, peroneus brevis, flexor digitorum longus, flexor hallucis longus, and tibialis posterior) and the dorsiflexors (tibialis anterior, extensor digitorum longus, and extensor hallucis longus) were identified. A representative image of upper leg ROIs drawn on a CT scan, a corresponding PET image, and a co-registration of the CT and PET images is displayed in Figure 2. The PET images were acquired immediately after the treadmill task and, therefore, the glucose uptake values closely reflected the uptake of FDG during walking. Standardized uptake values (SUV) based on the injected dose and body weight were calculated for each muscle. Although the participants fasted for a minimum of 6 h in order to minimize the impact, SUVs may be affected by blood glucose and insulin levels. Therefore, the SUV data were analyzed without normalization and as values normalized to standard blood glucose (adjusted to 100 mg/dL) [54,55], and/or the liver as a reference tissue [56,57,58], to allow for comparison across experimental sessions. The data were analyzed using PMOD Version 4.001 (PMD Technologies LLC, Zürich, Switzerland). The more-affected (weaker) legs were assigned using the knee extensor torque data obtained in Session 1. Asymmetry indices (AIs) were calculated to determine the magnitude of the asymmetry in SUVs between the legs using a previously used equation (Equation (1)) [59,60,61]. An AI greater than 10% was considered asymmetric [59,62].

(1) $$\frac{\left[less-affected\;side\;strength\;\left(Nm\right)]-[more-affected\;side\;strength\;\left(Nm\right)\right]}{\left(0.5\right)\left[less-affected\;side\;strength\;\left(Nm\right)]+[more-affected\;strength\;\left(Nm\right)\right]}\times 100$$

### 2.7. Statistical Analysis

In order to compare muscle activity between the legs and experimental sessions, SUVs were averaged for each muscle group (knee extensors, knee flexors, plantar flexors, and dorsiflexors). The assumptions for normality were investigated via histograms, Q-Q plots, and the Shapiro-Wilk test, but were not met. Therefore, nonparametric paired tests (Wilcoxon tests) were performed on the normalized data to compare the muscle groups of the left and right leg within each condition (e.g., left vs. right knee extensors during the tDCS condition) and to compare the muscle groups of each leg between conditions (e.g., left knee extensors in Sham vs. tDCS). Significance was accepted at *p* < 0.05. Analyses were performed using GraphPad Prism 8.1.2 (GraphPad Software, San Diego, CA, USA).

## 3. Results

The participants completed all of the testing conditions and no data were missing or removed. Subject 1 was able to correctly indicate which stimulation condition (tDCS or Sham) they experienced in each session, while Subject 2 was unable to differentiate between the conditions. Table 1 summarizes the subject characteristics. Height, weight, BMI, and Patient Determined Disease Steps (PDDS) scores were similar for both participants. However, age (Subject 1 = 56, Subject 2 = 44), time since diagnosis (Subject 1 = 20 years, Subject 2 = 15 years), and physical activity levels differed between the subjects. Both subjects had concomitant medication intake, consisting of immunomodulating (Subject 1 and 2), anti-fatigue (Subject 1), antidepressant (Subject 1), antiepileptic (Subject 1), and walking (Subject 1) agents. Pharmacological treatment was stable throughout the experimental protocol.

### 3.1. Clinical Case Description—Case 1

**Figure 3:**
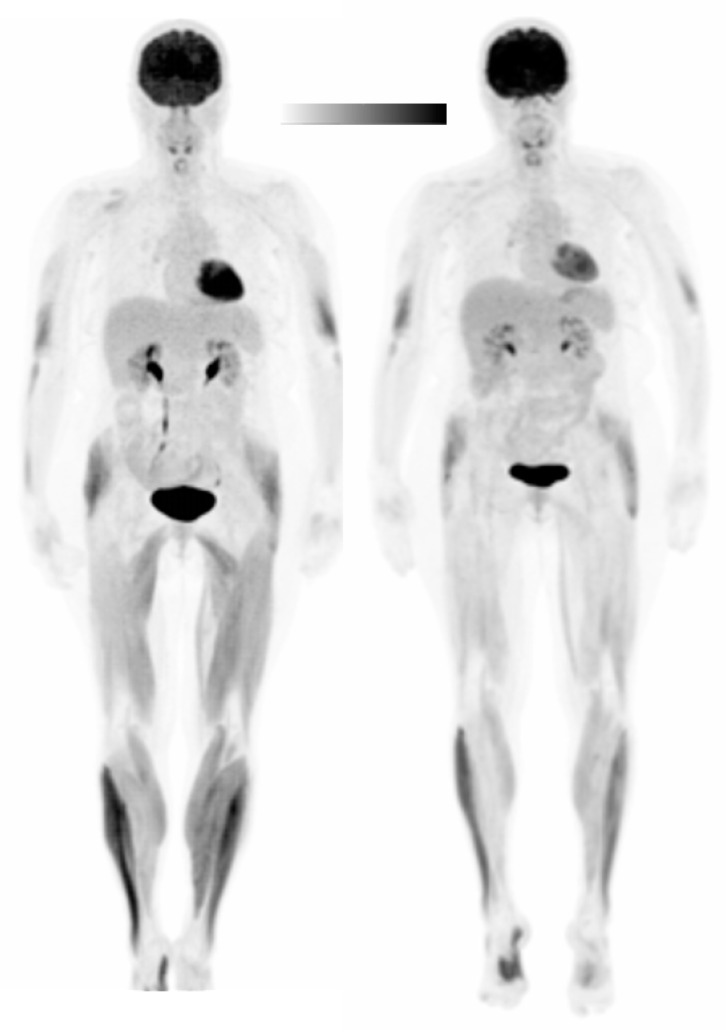
Whole-body positron emission tomography (PET) image of Subject 1 in the Sham (**left**) and tDCS (**right**) conditions. The Sham is as displayed. A scalar was applied to the tDCS image to normalize to the liver standardized uptake values (SUVs). Black denotes the greatest signal intensity, followed by gray, then white.

In Subject 1, peak torque was 43 Nm and 54 Nm for the left and right knee extensors, respectively. Therefore, the anode was placed over the right M1 (C4) to stimulate the area of the cortex area corresponding to the left leg. tDCS was well-tolerated and burning, tingling, and buzzing sensations were reported with mild to moderate severity (range from 1–6 on a Likert-type severity scale). Treadmill speed was 1.5 mph during both experimental sessions. During the walking task, an RPE range of 0.5–5 was reported in the Sham session and an RPE range of 0.5–3 was reported in the tDCS session. An RPE of 3 was first reported at minute 8 in the Sham session and at minute 13 in the tDCS session. Blood glucose concentration was 70 mg/dL in Session 1 (Sham) and 82 mg/dL in Session 2 (tDCS) and liver SUV_mean_ was 1.90 g/mL in Session 1 and 2.01 g/mL in Session 2. Table 2 presents glucose uptake (SUV) normalized to a standard blood glucose (NG) level of 100 mg/dL [54,55] and to the liver (NL) [57,58]. Statistical findings were identical for both normalization methods. SUVs significantly decreased in the tDCS condition in the left knee flexors (*p* = 0.031), left upper leg (*p* = 0.002), left (*p* = 0.016) and right (*p* = 0.016) plantar flexors, and the left (*p* = 0.002) and right (*p* = 0.002) lower leg. SUVs were significantly different between the left and right legs in the knee flexors in the tDCS condition (*p* = 0.031) and in the upper leg in the Sham condition (*p* = 0.027). Figure 3 provides a visual image of the SUV differences between the muscle groups in the Sham (left) and tDCS (right) conditions. In Sham, glucose uptake was asymmetric (AI > 10%) in all muscle groups. However, after tDCS the knee extensors (Sham AI = −31.64%, tDCS AI = 2.76%), upper leg (Sham AI = −34.95%, tDCS AI = −6.29%) and dorsiflexors (Sham AI = 12.63%, AI = −1.91%) had symmetric glucose uptake. AI values after Sham and tDCS and the difference in AIs between conditions are reported in Table 3.

### 3.2. Clinical Case Description—Case 2

**Figure 4:**
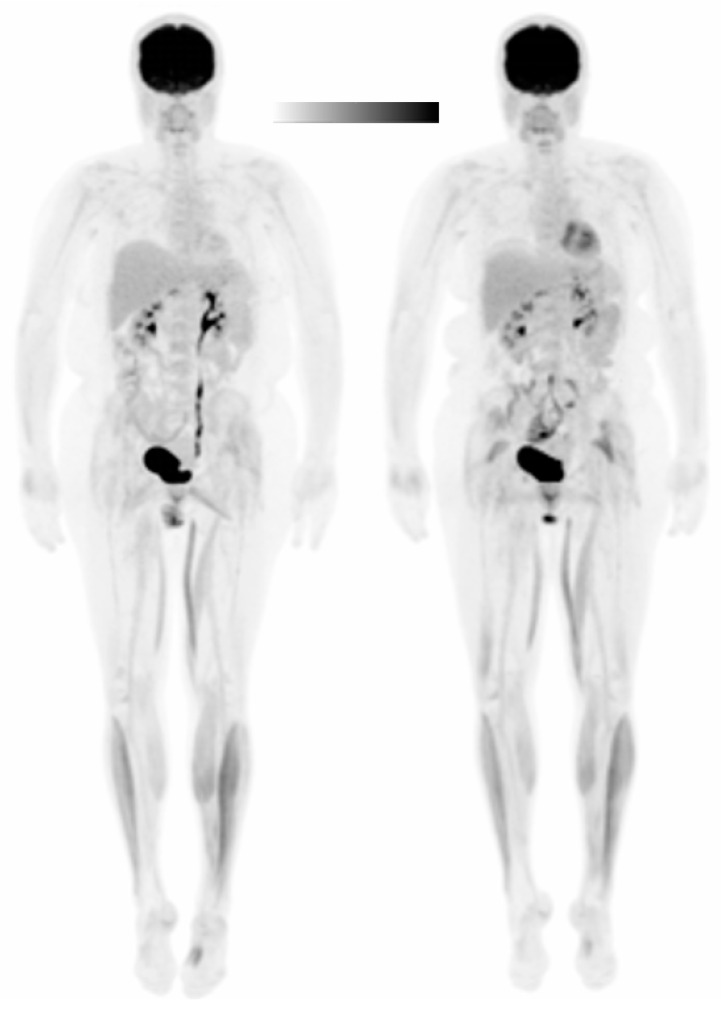
Whole-body positron emission tomography (PET) image of Subject 2 in the Sham (**left**) and tDCS (**right**) conditions. The Sham is as displayed. A scalar was applied to the tDCS image to normalize to the liver SUVs. Black denotes the greatest signal intensity, followed by gray, then white.

Subject 2 self-reported participating in vigorous physical activity at least three times a week for the last eight years. Their peak torque was 57 Nm and 48 Nm for the left and right knee extensors, respectively. Therefore, the anode was placed over the left M1 (C3) to stimulate the area of the cortex area corresponding to the right leg. tDCS was well-tolerated and burning, tingling, itching, and prickling sensations were reported with mild to moderate severity (range from 1–5 on a Likert-type severity scale). Treadmill speed was 2.0 mph during both experimental sessions. During the walking task, an RPE range of 1–3 was reported for the Sham session and an RPE range of 0.5–3 was reported for the tDCS session. An RPE of 3 was first reported at minute 19 in the Sham condition and minute 16 in the tDCS condition. Blood glucose concentration was similar in both sessions (Session 1 = 81 mL/dL, Session 2 = 83 mL/dL). Liver SUV_mean_ was 2.05 g/mL in Session 1 and 1.86 g/mL in Session 2. Table 4 presents glucose uptake (SUV) normalized to the liver (NL) ROI [56,57,58]. SUVs were not normalized to blood glucose because concentrations were similar across experimental sessions. SUVs were significantly increased in tDCS compared to Sham in the left (*p* = 0.014) and right (*p* = 0.006) upper legs, right plantar flexors (*p* = 0.016), and right lower leg (*p* = 0.009). Figure 4 provides a visual image of the SUV differences between the muscle groups in the Sham (left) and tDCS (right) conditions. In the Sham condition, glucose uptake in the knee extensors was the only muscle group considered symmetric (AI = −5.89%). There was no change in asymmetry status (i.e., asymmetric to symmetric or symmetric to asymmetric) in any of the muscle groups after tDCS. AI values after Sham and tDCS and the difference in AIs between conditions are reported in Table 5.

## 4. Discussion

To our knowledge, this is the first study that utilized PET with FDG to investigate changes in glucose metabolism and asymmetries in lower limb skeletal muscles after a single session of tDCS in PwMS. The main findings of this study were that tDCS (1) decreased FDG uptake in the left knee flexors, left upper leg, plantar flexors, and lower legs and improved asymmetries in the knee extensors, upper leg and dorsiflexors in Subject 1, and (2) increased FDG uptake in the upper legs, right plantar flexors, and right lower leg with no effect on asymmetries in Subject 2.

Subject 1 was a 56-year-old woman with MS (20 years since diagnosis) who was moderately disabled (PDDS score = 3) and self-reported as not physically active. After tDCS, she showed reduced glucose uptake (GU) in leg muscles and more symmetric GU in the knee extensors and dorsiflexors compared to Sham. Furthermore, her peak RPE score and the rate of change in RPE were lower in the tDCS condition. Many mildly impaired PwMS tend to live sedentary lifestyles, have low cardiovascular fitness, an altered muscle fiber distribution typical of disuse, and poor exercise tolerance, which may increase the energy demand of activities like walking [63,64,65,66]. Consequently, fatigue and reduced mobility are key problems in PwMS. As previously discussed, tDCS over the motor cortex may increase cortical excitability and enhance the neural drive from the cortex to the spinal motor neuron pool. It is feasible that the excitatory effects of anodal tDCS would be more likely to occur in patients with diseases characterized by hypo-excitability of motor areas. This premise is in line with recent studies reporting no effect of tDCS on physical performances in healthy subjects [67,68,69]. In the current study, it is proposed that tDCS was able to increase the cortical excitability of impaired motor areas in Subject 1, resulting in a more effective muscle activation strategy with decreased energy demands, decreased uptake asymmetry, and prolonged performance fatigability resistance. In PwMS, it is suggested that some muscles, particularly those on the less-affected (stronger) leg are minimally affected or unaffected by MS. This may explain why some muscles were not responsive to tDCS in Subject 1, indicating a ceiling effect of tDCS on cortical excitability. In other words, there may be a limited capacity to improve muscle activation strategies in healthy muscles compared to those with motor impairment. Lastly, Subject 1 was able to correctly identify the stimulation condition (tDCS or Sham) in each session and, therefore, placebo effects cannot be dismissed. Difficulty with blinding maintenance is common in tDCS [70,71] and should be addressed in future study designs.

Clinical case study 2 was a 44-year-old woman with MS (15 years since diagnosis) who was also moderately disabled (PDDS score = 3). Importantly, she self-reported as physically active. After tDCS, she showed higher GU values in the upper and lower leg muscles and had no changes in muscle symmetry status (i.e., asymmetric to symmetric) compared to Sham. There was no change in the peak RPE score reported between conditions, but RPE peaked sooner after tDCS (at minute 16) compared to the Sham condition (at minute 19). Compared to Subject 1, it seems that Subject 2 was less affected by MS, potentially due to her higher habitual physical activity, her younger age, and the shorter time since diagnosis. Although both subjects reported the same PDDS score, it should be noted that the PDDS, as well the Expanded Disability Status Scale (EDSS), are often insensitive to subtle functional impairments and, therefore, makes walking impairments at an early disease stage difficult to detect [72]. Moreover, differences in the cortical excitation/inhibition (E/I) balance was potentially different between the subjects at baseline [73]. Therefore, it is possible that the effect of tDCS on the E/I balance may have been optimal for Subject 1 but non-optimal for Subject 2 [73]. Subject 2 may have had fewer motor areas with hypo-excitability and may be more comparable to healthy subjects. Indeed, when tDCS was applied to the motor cortex of healthy subjects, performance fatigability increased [74,75], which indicated that tDCS may induce hyperexcitability in certain motor areas, resulting in neuronal activity that may hamper endogenous signals [76,77]. This may explain, at least in part, the higher RPE and higher SUVs after tDCS in Subject 2, which indicates greater energy demands and an earlier onset of performance fatigability.

Interestingly, tDCS affected both the less- and more-affected legs of Subject 1. One reason might be that the relatively large tDCS electrode covered more areas than the M1 representation of the more-affected leg. Furthermore, it is also very likely that tDCS effects on the left hemisphere may influence the right hemisphere, consistent with previous studies [78,79,80,81]. For example, the results of Mondini et al. [78] indicated effects of tDCS on spectral EEG power on the side contralateral to stimulation, and Park et al. [79] found that stimulation of the left dorsolateral prefrontal cortex had diffuse effects on right hemisphere areas. The present finding also supports the idea of tDCS affecting interhemispheric cooperation of the primary motor cortices during motor performance [79] and may also help explain the promising findings of this case study.

There are some limitations to this study. The small number of participants in this case study limits the generalizability of the results. Although PET and FDG allowed for the examination of muscle activation strategies, the low temporal resolution of this technique is a limitation. It is possible that muscle activation strategies were more symmetric at the beginning of the task and shifted to a more asymmetric strategy as the task progressed (or vice versa), but such a determination was not feasible with the current technique. Although the location and size of the anode allowed for the M1 leg area to be covered and potentially stimulated [51], differences in head size may have resulted in inconsistent stimulation of the leg areas between the subjects. Similarly, anatomical differences (e.g., skull thickness, cerebrospinal fluid thickness, and variations in cortical morphology) were not assessed and may have resulted in variations in the electric field magnitude that reached the cortex [82,83,84]. Because Subject 1 was not successfully blinded from stimulation condition, the results should be interpreted with caution. Moreover, we did not examine how walking performance was affected by alterations in glucose uptake using clinical walking assessments (e.g., 25 ft walk test, 6-min walk test, etc.) and the results are not generalizable to other walking intensities or tasks of daily living, such as stair climbing. Although the explanation of our results was largely based on the physical activity levels of the participants, objective measures of physical activity were not obtained.

Future larger studies should objectively measure the influence of physical activity levels and disease severity on glucose metabolism and walking performance using clinical assessments after tDCS. Furthermore, MU recruitment strategies during force production could be further investigated with high density multichannel EMG. In addition, other variables, such as age and sex, should also be explored and accounted for in these studies. Lastly, the effects and duration of other tDCS parameters, such as stimulation intensity, alternative Sham protocols, and the number of tDCS sessions, on glucose metabolism in PwMS should also be investigated. Specifically, repeated sessions of tDCS have had promising preliminary results [85] and may induce additive effects [86].

## 5. Conclusions

For one physically inactive subject, tDCS decreased GU and improved lower limb asymmetries during walking, which suggest that tDCS may have increased neural drive to the muscle and lowered energetic demands and perceived exertion. For one physically active subject, the purported increased neural drive resulted in a contrary finding of increased GU and higher energy demands. Thus, the effects of tDCS in PwMS might be mediated by habitual physical activity and inactive subjects might experience more benefit from the stimulation.

## Figures and Tables

**Table 1:** Subject characteristics.

Subject	Sex	Age (years)	Height (cm)	Weight (kg)	BMI	PDDS	Time Since Diagnosis	Physically Active *
**Subject 1**	F	56	158	59	23.6	3.0	20 years	No
**Subject 2**	F	44	155	62	25.81	3.0	15 years	Yes

F, Female; BMI, Body Mass Index; PPDS, Patient Determined Disease Steps. * Subjects were considered physically active if they self-reported participating in at least 30 min of moderate-intensity physical activity on at least three days of the week for the last three months.

**Table 2:** Glucose standardized uptake values (SUV in g/mL) in individual muscles, muscle group averages, and upper and lower leg averages after Sham/tDCS and walking in Subject 1. The left leg was objectively determined to be the weaker lower limb for this subject. Non-normalized (NN) SUVs and SUVs normalized to standard blood glucose (NG, adjusted to 100 mg/dL) and to the liver (NL) are reported. Data are Volume of Interest (VOI) mean ± standard deviation of glucose uptake within the muscle. Rows in bold represent the mean and standard deviation of the respective muscle groups.

	NN Left Leg		NN Right Leg		NG Left Leg		NG Right Leg		NL Left Leg		NL Right Leg	
Muscle Group	Sham	tDCS	Sham	tDCS	Sham	tDCS	Sham	tDCS	Sham	tDCS	Sham	tDCS
**Knee Extensors**												
Rectus Femoris	0.76 ± 0.38	0.63 ± 0.22	0.64 ± 0.27	0.55 ± 0.20	0.76 ± 0.38	0.54 ± 0.19	0.64 ± 0.27	0.47 ± 0.17	0.40 ± 0.20	0.31 ± 0.11	0.34 ± 0.14	0.27 ± 0.10
Vastus Medialis	2.45 ± 0.97	1.16 ± 0.39	2.02 ± 0.80	1.34 ± 0.48	2.45 ± 0.97	0.99 ± 0.33	2.02 ± 0.80	1.14 ± 0.41	1.29 ± 0.51	0.58 ± 0.19	1.07 ± 0.42	0.67 ± 0.24
Vastus Intermedius	2.44 ± 0.47	1.26 ± 0.48	1.67 ± 0.43	1.34 ± 0.39	2.44 ± 0.47	1.08 ± 0.41	1.67 ± 0.43	1.14 ± 0.33	1.29 ± 0.25	0.63 ± 0.24	0.88 ± 0.23	0.67 ± 0.19
Vastus Lateralis	1.87 ± 0.59	1.01 ± 0.38	1.13 ± 0.42	0.95 ± 0.33	1.87 ± 0.59	0.86 ± 0.32	1.13 ± 0.42	0.81 ± 0.28	0.98 ± 0.31	0.50 ± 0.19	0.60 ± 0.22	0.47 ± 0.17
**Mean ± SD**	**1.88 ± 0.80**	**1.02 ± 0.28**	**1.37 ± 0.61**	**1.05 ± 0.38**	**1.88 ± 0.80**	**0.87 ± 0.24**	**1.37 ± 0.61**	**0.89 ± 0.32**	**0.99 ± 0.42**	**0.51 ± 0.14**	**0.72 ± 0.32**	**0.52 ± 0.19**
**Knee Flexors**												
BF—Long Head	0.54 ± 0.21	0.56 ± 0.26	0.98 ± 0.28	0.56 ± 0.16	0.54 ± 0.21	0.48 ± 0.22	0.98 ± 0.28	0.47 ± 0.14	0.28 ± 0.11	0.28 ± 0.13	0.52 ± 0.15	0.28 ± 0.08
BF—Short Head	2.02 ± 0.62	1.68 ± 0.59	0.46 ± 0.11	1.17 ± 0.43	2.02 ± 0.62	1.43 ± 0.50	0.46 ± 0.11	1.00 ± 0.37	1.07 ± 0.33	0.84 ± 0.29	0.24 ± 0.06	0.58 ± 0.22
Semimembranosus	0.55 ± 0.15	0.57 ± 0.13	0.45 ± 0.10	0.52 ± 0.10	0.55 ± 0.15	0.49 ± 0.11	0.45 ± 0.10	0.45 ± 0.09	0.29 ± 0.08	0.28 ± 0.06	0.24 ± 0.06	0.26 ± 0.05
Semitendinosus	0.56 ± 0.21	0.53 ± 0.12	0.48 ± 0.13	0.52 ± 0.15	0.56 ± 0.21	0.45 ± 0.11	0.48 ± 0.13	0.44 ± 0.13	0.29 ± 0.11	0.27 ± 0.06	0.26 ± 0.07	0.26 ± 0.07
Sartorius	0.74 ± 0.33	0.59 ± 0.27	0.53 ± 0.24	0.58 ± 0.21	0.74 ± 0.33	0.51 ± 0.23	0.53 ± 0.24	0.49 ± 0.18	0.39 ± 0.17	0.29 ± 0.14	0.28 ± 0.12	0.29 ± 0.10
Gracilis	1.27 ± 0.51	1.11 ± 0.47	0.53 ± 0.33	0.51 ± 0.14	1.27 ± 0.51	0.95 ± 0.40	0.53 ± 0.33	0.44 ± 0.12	0.67 ± 0.27	0.55 ± 0.23	0.28 ± 0.18	0.25 ± 0.07
**Mean ± SD**	**0.94 ± 0.60**	**0.84 ± 0.47**	**0.58 ± 0.22**	**0.64 ± 0.26**	**0.94 ± 0.60 ^±^**	0.72 ± 0.40 *	**0.58 ± 0.22**	**0.55 ± 0.22**	**0.50 ± 0.31 ^±^**	0.72 ± 0.40 *	**0.30 ± 0.11**	**0.32 ± 0.13**
**Upper Leg Mean ± SD**	**1.32 ± 0.80**	**0.91 ± 0.39**	**0.89 ± 0.56**	**0.80 ± 0.36**	1.32 ± 0.80 ^±^ *	**0.78 ± 0.34**	**0.89 ± 0.56**	**0.68 ± 0.30**	0.69 ± 0.42 ^±^ *	**0.78 ± 0.34**	**0.47 ± 0.29**	**0.40 ± 0.18**
**Plantar Flexors**												
Gastrocnemius	2.74 ± 0.98	1.53 ± 0.75	2.45 ± 0.67	1.47 ± 0.70	2.74 ± 0.98	1.31 ± 0.64	2.45 ± 0.67	1.26 ± 0.59	1.44 ± 0.52	0.76 ± 0.37	1.31 ± 0.35	0.73 ± 0.35
Soleus	3.59 ± 0.92	1.65 ± 0.34	1.98 ± 0.66	1.21 ± 0.30	3.59 ± 0.92	1.41 ± 0.29	1.98 ± 0.66	1.03 ± 0.26	1.89 ± 0.48	0.82 ± 0.17	1.04 ± 0.35	0.60 ± 0.15
Peroneus Longus	1.84 ± 0.73	1.38 ± 0.55	5.08 ± 1.98	4.10 ± 2.18	1.84 ± 0.73	1.18 ± 0.47	5.08 ± 1.98	3.50 ± 1.86	0.97 ± 0.39	0.69 ± 0.28	2.68 ± 1.04	2.04 ± 1.09
Peroneus Brevis	1.64 ± 0.86	1.36 ± 0.34	4.62 ± 1.98	3.92 ± 1.90	1.64 ± 0.86	1.16 ± 0.29	4.62 ± 1.98	3.34 ± 1.62	0.86 ± 0.46	0.68 ± 0.17	2.43 ± 1.04	1.95 ± 0.94
Flexor Digitorum	2.64 ± 1.51	1.94 ± 0.52	1.94 ± 0.46	1.27 ± 0.37	2.64 ± 1.51	1.65 ± 0.44	1.94 ± 0.46	1.08 ± 0.32	1.39 ± 0.79	0.96 ± 0.26	1.02 ± 0.24	0.63 ± 0.19
Flexor Hallucis	2.44 ± 1.47	1.66 ± 0.42	1.64 ± 0.61	1.19 ± 0.21	2.44 ± 1.47	1.42 ± 0.36	1.64 ± 0.61	1.01 ± 0.18	1.28 ± 0.77	0.83 ± 0.21	0.86 ± 0.32	0.59 ± 0.10
Tibialis Posterior	1.22 ± 0.89	1.13 ± 0.56	1.21 ± 0.69	1.07 ± 0.41	1.22 ± 0.89	0.97 ± 0.48	1.21 ± 0.69	0.91 ± 0.35	0.65 ± 0.47	0.56 ± 028	0.64 ± 0.36	0.53 ± 0.20
**Mean ± SD**	**2.30 ± 0.79**	**1.52 ± 0.26**	**2.71 ± 1.52**	**2.03 ± 1.36**	**2.30 ± 0.79 ^±^**	**1.30 ± 0.22**	**2.71 ± 1.52 ^±^**	**1.73 ± 1.16**	**1.21 ± 0.42 ^±^**	**0.76 ± 0.13**	**1.43 ± 0.80 ^±^**	**1.01 ± 0.68**
**Dorsiflexors**												
Tibialis Anterior	5.31 ± 1.80	3.05 ± 1.66	4.36 ± 1.51	1.60 ± 0.81	5.31 ± 1.80	2.60 ± 1.41	4.36 ± 1.51	1.37 ± 0.69	2.80 ± 0.95	1.52 ± 0.82	2.30 ± 0.80	0.80 ± 0.40
Extensor Digitorum	3.37 ± 1.63	2.10 ± 1.18	5.49 ± 2.32	3.93 ± 2.14	3.37 ± 1.63	1.80 ± 1.00	5.49 ± 2.32	3.35 ± 1.83	1.78 ± 0.86	1.05 ± 0.59	2.89 ± 1.22	1.96 ± 1.07
Extensor Hallucis	2.44 ± 1.47	3.31 ± 1.02	5.34 ± 1.99	2.77 ± 1.48	2.44 ± 1.47	2.82 ± 0.87	5.34 ± 1.99	2.36 ± 1.26	2.48 ± 0.64	1.65 ± 0.51	2.81 ± 1.05	1.38 ± 0.74
**Mean ± SD**	**4.46 ± 0.99**	**2.82 ± 0.64**	**5.07 ± 0.62**	**2.77 ± 1.67**	**4.46 ± 0.99**	**2.41 ± 0.54**	**5.07 ± 0.62**	**2.36 ± 0.99**	**2.35 ± 0.52**	**1.40 ± 0.32**	**2.67 ± 0.32**	**1.38 ± 0.58**
**Lower Leg Mean ± SD**	**2.95 ± 1.32**	**1.91 ± 0.73**	**3.42 ± 1.71**	**2.25 ± 1.29**	**2.95 ± 1.32 ^±^**	**1.63 ± 0.62**	**3.42 ± 1.71 ^±^**	**1.92 ± 1.10**	**1.55 ± 0.69 ^±^**	**0.95 ± 0.36**	**1.80 ± 0.90 ^±^**	**1.12 ± 0.64**

^±^ Indicates significantly different from tDCS. * Indicates significantly different from the other leg in the same condition (Sham or tDCS). Significance was accepted at *p* < 0.05.

**Table 3:** Glucose standardized uptake value (SUV) asymmetry indices (AIs) after Sham and tDCS in Subject 1 and the difference between conditions. Negative values indicate a decrease in symmetry after tDCS. An AI greater than 10% was considered asymmetric. Symmetric values are in bold.

Muscle Group	Sham	tDCS	ΔAI (%)
Knee Extensors	−31.64%	**2.57%**	34.21
Knee Flexors	−49.81%	−16.32%	24.49
Upper Leg	−34.95%	**−6.29%**	28.65
Plantar Flexors	16.22%	28.67%	−12.45
Dorsiflexors	12.63%	**−1.91%**	14.54
Lower Leg	14.61%	16.36%	−1.76

**Table 4:** Glucose standardized uptake values (SUV in g/mL) in individual muscles, muscle group averages, and upper and lower leg averages after Sham/tDCS and walking in Subject 2. The right leg was objectively determined to be the weaker lower limb for this subject. Non-normalized (NN) SUVs and SUVs normalized to standard blood glucose (NG, adjusted to 100 mg/dL) and to the liver (NL) are reported. Data are Volume of Interest (VOI) mean ± standard deviation of glucose uptake within the muscle. Rows in bold represent the mean and standard deviation of the respective muscle groups.

	NN Left Leg		NN Right Leg		NL Left Leg		NL Right Leg	
Muscle Group	Sham	tDCS	Sham	tDCS	Sham	tDCS	Sham	tDCS
**Knee Extensors**								
Rectus Femoris	0.54 ± 0.11	0.64 ± 0.08	0.51 ± 0.11	0.62 ± 0.09	0.26 ± 0.05	0.35 ± 0.05	0.25 ± 0.05	0.34 ± 0.05
Vastus Medialis	0.61 ± 0.17	0.63 ± 0.12	0.58 ± 0.17	0.59 ± 0.11	0.30 ± 0.08	0.34 ± 0.06	0.28 ± 0.08	0.32 ± 0.06
Vastus Intermedius	0.58 ± 0.09	0.67 ± 0.09	0.53 ± 0.07	0.60 ± 0.08	0.28 ± 0.04	0.36 ± 0.05	0.26 ± 0.03	0.32 ± 0.04
Vastus Lateralis	0.53 ± 0.10	0.57 ± 0.08	0.51 ± 0.10	0.53 ± 0.07	0.26 ± 0.05	0.30 ± 0.05	0.25 ± 0.05	0.29 ± 0.04
**Mean ± SD**	**0.56 ± 0.04**	**0.63 ± 0.04**	**0.53 ± 0.03**	**0.58 ± 0.04**	**0.27 ± 0.02**	**0.34 ± 0.02**	**0.26 ± 0.02**	**0.32 ± 0.02**
**Knee Flexors**								
BF—Long Head	0.50 ± 0.09	0.48 ± 0.08	0.52 ± 0.10	0.54 ± 0.12	0.24 ± 0.04	0.26 ± 0.04	0.25 ± 0.05	0.29 ± 0.07
BF—Short Head	0.81 ± 0.19	0.91 ± 0.26	0.77 ± 0.19	1.19 ± 0.43	0.39 ± 0.09	0.49 ± 0.14	0.38 ± 0.09	0.64 ± 0.23
Semimembranosus	0.58 ± 0.28	0.54 ± 0.24	0.64 ± 0.14	0.54 ± 0.08	0.28 ± 0.14	0.29 ± 0.13	0.31 ± 0.07	0.29 ± 0.04
Semitendinosus	0.59 ± 0.09	0.59 ± 0.09	0.50 ± 0.09	0.55 ± 0.10	0.29 ± 0.05	0.32 ± 0.05	0.24 ± 0.05	0.30 ± 0.05
Sartorius	1.46 ± 0.54	1.49 ± 0.61	0.66 ± 0.18	0.62 ± 0.11	0.71 ± 0.26	0.80 ± 0.33	0.32 ± 0.09	0.33 ± 0.06
Gracilis	1.68 ± 1.16	1.46 ± 0.87	0.96 ± 0.54	1.10 ± 0.57	0.82 ± 0.57	0.79 ± 0.47	0.47 ± 0.26	0.59 ± 0.31
**Mean ± SD**	**0.94 ± 0.51**	**0.91 ± 0.46**	**0.67 ± 0.17**	**0.76 ± 0.31**	**0.46 ± 0.25**	**0.49 ± 0.25**	**0.33 ± 0.08**	**0.41 ± 0.16**
**Upper Leg Mean ± SD**	**0.79 ± 0.42**	**0.80 ± 0.38**	**0.62 ± 0.15**	**0.69 ± 0.31**	0.38 ± 0.21 * ^±^	**0.43 ± 0.20**	**0.30 ± 0.07 ^±^**	**0.37 ± 0.13**
**Plantar Flexors**								
Gastrocnemius	1.40 ± 0.69	1.26 ± 0.51	0.93 ± 0.37	1.05 ± 0.35	0.68 ± 0.34	0.68 ± 0.27	0.45 ± 0.18	0.57 ± 0.19
Soleus	1.33 ± 0.44	1.08 ± 0.21	0.86 ± 0.22	0.91 ± 0.15	0.65 ± 0.22	0.58 ± 0.11	0.42 ± 0.11	0.49 ± 0.08
Peroneus Longus	1.27 ± 0.32	1.83 ± 0.53	0.83 ± 0.24	0.94 ± 0.17	0.62 ± 0.16	0.99 ± 0.29	0.41 ± 0.12	0.51 ± 0.09
Peroneus Brevis	1.15 ± 0.23	0.98 ± 0.28	0.85 ± 0.13	0.90 ± 0.18	0.56 ± 0.11	0.53 ± 0.15	0.42 ± 0.06	0.48 ± 0.10
Flexor Digitorum	1.40 ± 0.36	1.15 ± 0.22	1.03 ± 0.22	1.01 ± 0.16	0.68 ± 0.17	0.62 ± 0.12	0.50 ± 0.11	0.54 ± 0.09
Flexor Hallucis	1.45 ± 0.20	1.06 ± 0.21	0.91 ± 0.13	1.94 ± 0.35	0.71 ± 0.10	0.57 ± 0.11	0.44 ± 0.06	0.54 ± 0.12
Tibialis Posterior	1.07 ± 0.40	0.87 ± 0.34	1.01 ± 0.29	0.97 ± 0.32	0.52 ± 0.19	0.47 ± 0.18	0.49 ± 0.14	0.52 ± 0.17
**Mean ± SD**	**1.29 ± 0.14**	**1.18 ± 0.31**	**0.92 ± 0.08**	**1.10 ± 0.37**	0.63 ± 0.07 *	**0.63 ± 0.17**	**0.45 ± 0.04 ^±^**	**0.52 ± 0.03**
**Dorsiflexors**								
Tibialis Anterior	3.55 ± 1.03	2.84 ± 0.79	2.59 ± 0.71	2.24 ± 0.54	1.73 ± 0.50	1.53 ± 0.42	1.26 ± 0.35	1.20 ± 0.29
Extensor Digitorum	1.17 ± 0.29	1.77 ± 0.56	0.87 ± 0.35	1.57 ± 0.49	0.57 ± 0.14	0.95 ± 0.30	0.42 ± 0.17	0.85 ± 0.26
Extensor Hallucis	2.11 ± 0.70	2.03 ± 0.51	1.90 ± 0.56	1.94 ± 0.35	1.03 ± 0.34	1.09 ± 0.28	0.92 ± 0.27	1.04 ± 0.19
**Mean ± SD**	**2.28 ± 1.20**	**2.21 ± 0.56**	**1.79 ± 0.87**	**1.92 ± 0.33**	**1.11 ± 0.58**	**1.19 ± 0.30**	**0.87 ± 0.42**	**1.03 ± 0.18**
**Lower Leg Mean ± SD**	**1.59 ± 0.75**	**1.49 ± 0.62**	**1.18 ± 0.59**	**1.35 ± 0.52**	0.77 ± 0.36 *	0.80 ± 0.33 *	**0.57 ± 0.29 ^±^**	**0.68 ± 0.26**

^±^ Indicates significantly different from tDCS. * Indicates significantly different from the other leg in the same condition (Sham or tDCS). Significance was accepted at *p* < 0.05.

**Table 5:** Glucose standardized uptake value (SUV) asymmetry indices (AIs) after Sham and tDCS in Subject 2 and the difference between conditions. Negative values indicate a decrease in symmetry after tDCS. An AI greater than 10% was considered asymmetric. Symmetric values are in bold.

Muscle Group	Sham	tDCS	ΔAI
Knee Extensors	**−5.89%**	**−6.75%**	−0.86%
Knee Flexors	−24.03%	−15.09%	8.94%
Upper Leg	−18.82%	−11.80%	7.02%
Plantar Flexors	−42.15%	−22.80%	19.34%
Dorsiflexors	−23.98%	−14.42%	9.56%
Lower Leg	−34.36%	−19.06%	15.31%

## References

[1] Thompson A.J., Baranzini S.E., Geurts J., Hemmer B., Ciccarelli O. (2018). Multiple sclerosis. Lancet.

[2] Noseworthy J.H., Lucchinetti C., Rodriguez M., Weinshenker B.G. (2000). Multiple sclerosis. N. Engl. J. Med..

[3] Chaudhuri A. (2013). Multiple sclerosis is primarily a neurodegenerative disease. J. Neural Transm. (Vienna).

[4] Kindred J.H., Koo P.J., Rudroff T. (2014). Glucose uptake of the spinal cord in patients with multiple sclerosis detected by [18F]-Fluorodeoxyglucose PET/CT after walking. Spinal Cord.

[5] Schmierer K., Niehaus L., Roricht S., Meyer B.U. (2000). Conduction deficits of callosal fibres in early multiple sclerosis. J. Neurol. Neurosurg. Psychiatry.

[6] Delgado-Mendilivar J.M., Cadenas-Diaz J.C., Fernandez-Torrico J.M., Navarro-Mascarell G., Izquierdo G. (2005). A study of the quality of life in cases of multiple sclerosis. Rev. Neurol..

[7] Pfaffenberger N., Pfeiffer K.P., Deibl M., Hofer S., Gunther V., Ulmer H. (2006). Association of factors influencing health-related quality of life in MS. Acta Neurol. Scand..

[8] Confavreux C., Vukusic S., Moreau T., Adeleine P. (2000). Relapses and progression of disability in multiple sclerosis. N. Engl. J. Med..

[9] Frzovic D., Morris M.E., Vowels L. (2000). Clinical tests of standing balance: Performance of persons with multiple sclerosis. Arch. Phys. Med. Rehabil..

[10] Chung L.H., Remelius J.G., Van Emmerik R.E., Kent-Braun J.A. (2008). Leg power asymmetry and postural control in women with multiple sclerosis. Med. Sci. Sports Exerc..

[11] Workman C.D., Fietsam A.C., Rudroff T. (2020). Associations of lower limb joint asymmetry with fatigue and disability in people with multiple sclerosis. Clin. Biomech..

[12] Rudroff T., Proessl F. (2018). Effects of Muscle Function and Limb Loading Asymmetries on Gait and Balance in People With Multiple Sclerosis. Front. Physiol..

[13] Ketelhut N.B., Kindred J.H., Manago M.M., Hebert J.R., Rudroff T. (2015). Core muscle characteristics during walking of patients with multiple sclerosis. J. Rehabil. Res. Dev..

[14] Nitsche M.A., Paulus W. (2000). Excitability changes induced in the human motor cortex by weak transcranial direct current stimulation. J. Physiol..

[15] Nitsche M.A., Bikson M. (2017). Extending the parameter range for tDCS: Safety and tolerability of 4 mA stimulation. Brain Stimul..

[16] Tanaka S., Hanakawa T., Honda M., Watanabe K. (2009). Enhancement of pinch force in the lower leg by anodal transcranial direct current stimulation. Exp. Brain Res..

[17] Van Asseldonk E.H., Boonstra T.A. (2016). Transcranial Direct Current Stimulation of the Leg Motor Cortex Enhances Coordinated Motor Output During Walking With a Large Inter-Individual Variability. Brain Stimul..

[18] Cunningham D.A. (2017). Noninvasive brain stimulation enhances sustained muscle contractions by reducing neuromuscular fatigue: Implications for rehabilitation. J. Neurophysiol..

[19] Cuypers K., Leenus D.J., Van Wijmeersch B., Thijs H., Levin O., Swinnen S.P., Meesen R.L. (2013). Anodal tDCS increases corticospinal output and projection strength in multiple sclerosis. Neurosci. Lett..

[20] Jeffery D.T., Norton J.A., Roy F.D., Gorassini M.A. (2007). Effects of transcranial direct current stimulation on the excitability of the leg motor cortex. Exp. Brain Res..

[21] Kaminski E., Steele C.J., Hoff M., Gundlach C., Rjosk V., Sehm B., Villringer A., Ragert P. (2016). Transcranial direct current stimulation (tDCS) over primary motor cortex leg area promotes dynamic balance task performance. Clin. Neurophysiol..

[22] Workman C., Kamholz J., Rudroff T. (2019). Transcranial Direct Current Stimulation (tDCS) to Improve Gait in Multiple Sclerosis: A Timing Window Comparison. Front. Hum. Neurosci..

[23] Cogiamanian F., Marceglia S., Ardolino G., Barbieri S., Priori A. (2007). Improved isometric force endurance after transcranial direct current stimulation over the human motor cortical areas. Eur. J. Neurosci..

[24] Williams P.S., Hoffman R.L., Clark B.C. (2013). Preliminary evidence that anodal transcranial direct current stimulation enhances time to task failure of a sustained submaximal contraction. PLoS ONE.

[25] Lefaucheur J.P., Antal A., Ayache S.S., Benninger D.H., Brunelin J., Cogiamanian F., Cotelli M., De Ridder D., Ferrucci R., Langguth B. (2017). Evidence-based guidelines on the therapeutic use of transcranial direct current stimulation (tDCS). Clin. Neurophysiol..

[26] Iodice R., Dubbioso R., Ruggiero L., Santoro L., Manganelli F. (2015). Anodal transcranial direct current stimulation of motor cortex does not ameliorate spasticity in multiple sclerosis. Restor. Neurol. Neurosci..

[27] Oveisgharan S., Karimi Z., Abdi S., Sikaroodi H. (2019). The use of brain stimulation in the rehabilitation of walking disability in patients with multiple sclerosis: A randomized double-blind clinical trial study. Iran. J. Neurol..

[28] Pilloni G., Choi C., Coghe G., Cocco E., Krupp L.B., Pau M., Charvet L.E. (2020). Gait and Functional Mobility in Multiple Sclerosis: Immediate Effects of Transcranial Direct Current Stimulation (tDCS) Paired With Aerobic Exercise. Front. Neurol..

[29] Farina D., Merletti R., Enoka R.M. (2014). The extraction of neural strategies from the surface EMG: An update. J. Appl. Physiol. (1985).

[30] Farina D., Merletti R., Enoka R.M. (2004). The extraction of neural strategies from the surface EMG. J. Appl. Physiol. (1985).

[31] Rudroff T., Ketelhut N.B., Kindred J.H. (2018). Metabolic imaging in exercise physiology. J. Appl. Physiol. (1985).

[32] Rudroff T., Kindred J.H., Kalliokoski K.K. (2015). [18F]-FDG positron emission tomography—An established clinical tool opening a new window into exercise physiology. J. Appl. Physiol. (1985).

[33] Olgiati R., Burgunder J.M., Mumenthaler M. (1988). Increased energy cost of walking in multiple sclerosis: Effect of spasticity, ataxia, and weakness. Arch. Phys. Med. Rehabil..

[34] Olgiati R., Jacquet J., Di Prampero P.E. (1986). Energy cost of walking and exertional dyspnea in multiple sclerosis. Am. Rev. Respir. Dis..

[35] Agiovlasitis S., Motl R.W., Fernhall B. (2010). Prediction of oxygen uptake during level treadmill walking in people with multiple sclerosis. J. Rehabil. Med..

[36] Larson R.D., McCully K.K., Larson D.J., Pryor W.M., White L.J. (2013). Bilateral differences in lower-limb performance in individuals with multiple sclerosis. J. Rehabil. Res. Dev..

[37] Rudroff T., Kindred J.H., Koo P.J., Karki R., Hebert J.R. (2014). Asymmetric glucose uptake in leg muscles of patients with Multiple Sclerosis during walking detected by [18F]-FDG PET/CT. NeuroRehabilitation.

[38] Thompson A.J., Banwell B.L., Barkhof F., Carroll W.M., Coetzee T., Comi G., Correale J., Fazekas F., Filippi M., Freedman M.S. (2018). Diagnosis of multiple sclerosis: 2017 revisions of the McDonald criteria. Lancet Neurol..

[39] Learmonth Y.C., Motl R.W., Sandroff B.M., Pula J.H., Cadavid D. (2013). Validation of patient determined disease steps (PDDS) scale scores in persons with multiple sclerosis. BMC Neurol..

[40] Thompson P.D., Arena R., Riebe D., Pescatello L.S. (2013). ACSM’s new preparticipation health screening recommendations from ACSM’s guidelines for exercise testing and prescription, ninth edition. Curr. Sports Med. Rep..

[41] Nitsche M.A., Paulus W. (2001). Sustained excitability elevations induced by transcranial DC motor cortex stimulation in humans. Neurology.

[42] Batsikadze G., Moliadze V., Paulus W., Kuo M.F., Nitsche M.A. (2013). Partially non-linear stimulation intensity-dependent effects of direct current stimulation on motor cortex excitability in humans. J. Physiol..

[43] Boellaard R., Delgado-Bolton R., Oyen W.J., Giammarile F., Tatsch K., Eschner W., Verzijlbergen F.J., Barrington S.F., Pike L.C., Weber W.A. (2015). FDG PET/CT: EANM procedure guidelines for tumour imaging: Version 2.0. Eur. J. Nucl. Med. Mol. Imaging.

[44] Delbeke D., Coleman R.E., Guiberteau M.J., Brown M.L., Royal H.D., Siegel B.A., Townsend D.W., Berland L.L., Parker J.A., Hubner K. (2006). Procedure guideline for tumor imaging with 18F-FDG PET/CT 1.0. J. Nucl. Med..

[45] Samani M.M., Agboada D., Jamil A., Kuo M.F., Nitsche M.A. (2019). Titrating the neuroplastic effects of cathodal transcranial direct current stimulation (tDCS) over the primary motor cortex. Cortex.

[46] Ammann C., Spampinato D., Marquez-Ruiz J. (2016). Modulating Motor Learning through Transcranial Direct-Current Stimulation: An Integrative View. Front. Psychol..

[47] Kronberg G., Rahman A., Sharma M., Bikson M., Parra L.C. (2020). Direct current stimulation boosts hebbian plasticity in vitro. Brain Stimul..

[48] Santarnecchi E., Feurra M., Barneschi F., Acampa M., Bianco G., Cioncoloni D., Rossi A., Rossi S. (2014). Time Course of Corticospinal Excitability and Autonomic Function Interplay during and Following Monopolar tDCS. Front. Psychiatry.

[49] Workman C.D., Fietsam A.C., Rudroff T. (2020). Transcranial Direct Current Stimulation at 4 mA Induces Greater Leg Muscle Fatigability in Women Compared to Men. Brain Sci..

[50] Klem G.H., Luders H.O., Jasper H.H., Elger C. (1999). The ten-twenty electrode system of the International Federation. The International Federation of Clinical Neurophysiology. Electroencephalogr. Clin. Neurophysiol. Suppl..

[51] Foerster A.S., Rezaee Z., Paulus W., Nitsche M.A., Dutta A. (2018). Effects of Cathode Location and the Size of Anode on Anodal Transcranial Direct Current Stimulation Over the Leg Motor Area in Healthy Humans. Front. Neurosci..

[52] Jayaram G., Stinear J.W. (2009). The effects of transcranial stimulation on paretic lower limb motor excitability during walking. J. Clin. Neurophysiol..

[53] Foerster A., Yavari F., Farnad L., Jamil A., Paulus W., Nitsche M.A., Kuo M.F. (2019). Effects of electrode angle-orientation on the impact of transcranial direct current stimulation on motor cortex excitability. Brain Stimul..

[54] Sprinz C., Altmayer S., Zanon M., Watte G., Irion K., Marchiori E., Hochhegger B. (2018). Effects of blood glucose level on 18F-FDG uptake for PET/CT in normal organs: A systematic review. PLoS ONE.

[55] Sprinz C., Zanon M., Altmayer S., Watte G., Irion K., Marchiori E., Hochhegger B. (2018). Effects of blood glucose level on 18F fluorodeoxyglucose (18F-FDG) uptake for PET/CT in normal organs: An analysis on 5623 patients. Sci. Rep..

[56] Zasadny K.R., Wahl R.L. (1993). Standardized uptake values of normal tissues at PET with 2-[fluorine-18]-fluoro-2-deoxy-D-glucose: Variations with body weight and a method for correction. Radiology.

[57] Paquet N., Albert A., Foidart J., Hustinx R. (2004). Within-patient variability of (18)F-FDG: Standardized uptake values in normal tissues. J. Nucl. Med..

[58] Ramos C.D., Erdi Y.E., Gonen M., Riedel E., Yeung H.W., Macapinlac H.A., Chisin R., Larson S.M. (2001). FDG-PET standardized uptake values in normal anatomical structures using iterative reconstruction segmented attenuation correction and filtered back-projection. Eur. J. Nucl. Med..

[59] Proessl F., Ketelhut N.B., Rudroff T. (2018). No association of leg strength asymmetry with walking ability, fatigability, and fatigue in multiple sclerosis. Int. J. Rehabil. Res..

[60] Robinson R.O., Herzog W., Nigg B.M. (1987). Use of force platform variables to quantify the effects of chiropractic manipulation on gait symmetry. J. Manip. Physiol. Ther..

[61] Sadeghi H., Allard P., Prince F., Labelle H. (2000). Symmetry and limb dominance in able-bodied gait: A review. Gait Posture.

[62] Ithurburn M.P., Paterno M.V., Ford K.R., Hewett T.E., Schmitt L.C. (2015). Young Athletes With Quadriceps Femoris Strength Asymmetry at Return to Sport After Anterior Cruciate Ligament Reconstruction Demonstrate Asymmetric Single-Leg Drop-Landing Mechanics. Am. J. Sports Med..

[63] Waters R.L., Mulroy S. (1999). The energy expenditure of normal and pathologic gait. Gait Posture.

[64] White L.J., Dressendorfer R.H. (2004). Exercise and multiple sclerosis. Sports Med..

[65] Newman M.A., Dawes H., van den Berg M., Wade D.T., Burridge J., Izadi H. (2007). Can aerobic treadmill training reduce the effort of walking and fatigue in people with multiple sclerosis: A pilot study. Mult. Scler..

[66] Wens I., Dalgas U., Vandenabeele F., Krekels M., Grevendonk L., Eijnde B.O. (2014). Multiple sclerosis affects skeletal muscle characteristics. PLoS ONE.

[67] Hendy A.M., Kidgell D.J. (2013). Anodal tDCS applied during strength training enhances motor cortical plasticity. Med. Sci. Sports Exerc..

[68] Kan B., Dundas J.E., Nosaka K. (2013). Effect of transcranial direct current stimulation on elbow flexor maximal voluntary isometric strength and endurance. Appl. Physiol. Nutr. Metab..

[69] Lampropoulou S.I., Nowicky A.V. (2013). The effect of transcranial direct current stimulation on perception of effort in an isolated isometric elbow flexion task. Motor Control.

[70] Turi Z., Csifcsák G., Boayue N.M., Aslaksen P., Antal A., Paulus W., Groot J., Hawkins G.E., Forstmann B., Opitz A. (2019). Blinding is compromised for transcranial direct current stimulation at 1 mA for 20 min in young healthy adults. Eur. J. Neurosci..

[71] O’Connell N.E., Cossar J., Marston L., Wand B.M., Bunce D., Moseley G.L., De Souza L.H. (2012). Rethinking clinical trials of transcranial direct current stimulation: Participant and assessor blinding is inadequate at intensities of 2 mA. PLoS ONE.

[72] Martin C.L., Phillips B.A., Kilpatrick T.J., Butzkueven H., Tubridy N., McDonald E., Galea M.P. (2006). Gait and balance impairment in early multiple sclerosis in the absence of clinical disability. Mult. Scler..

[73] Krause B., Marquez-Ruiz J., Cohen Kadosh R. (2013). The effect of transcranial direct current stimulation: A role for cortical excitation/inhibition balance?. Front. Hum. Neurosci..

[74] Workman C.D., Kamholz J., Rudroff T. (2019). The Tolerability and Efficacy of 4 mA Transcranial Direct Current Stimulation on Leg Muscle Fatigability. Brain Sci..

[75] Workman C.D., Kamholz J., Rudroff T. (2020). Increased leg muscle fatigability during 2 mA and 4 mA transcranial direct current stimulation over the left motor cortex. Exp. Brain Res..

[76] Bastani A., Jaberzadeh S. (2013). Differential modulation of corticospinal excitability by different current densities of anodal transcranial direct current stimulation. PLoS ONE.

[77] Chai Z., Ma C., Jin X. (2019). Cortical stimulation for treatment of neurological disorders of hyperexcitability: A role of homeostatic plasticity. Neural Regen. Res..

[78] Mondini V., Mangia A.L., Cappello A. (2018). Single-session tDCS over the dominant hemisphere affects contralateral spectral EEG power, but does not enhance neurofeedback-guided event-related desynchronization of the non-dominant hemisphere’s sensorimotor rhythm. PLoS ONE.

[79] Park C.H., Chang W.H., Park J.Y., Shin Y.I., Kim S.T., Kim Y.H. (2013). Transcranial direct current stimulation increases resting state interhemispheric connectivity. Neurosci. Lett..

[80] Schambra H.M., Abe M., Luckenbaugh D.A., Reis J., Krakauer J.W., Cohen L.G. (2011). Probing for hemispheric specialization for motor skill learning: A transcranial direct current stimulation study. J. Neurophysiol..

[81] Waters S., Wiestler T., Diedrichsen J. (2017). Cooperation Not Competition: Bihemispheric tDCS and fMRI Show Role for Ipsilateral Hemisphere in Motor Learning. J. Neurosci..

[82] Opitz A., Paulus W., Will S., Antunes A., Thielscher A. (2015). Determinants of the electric field during transcranial direct current stimulation. Neuroimage.

[83] Datta A., Truong D., Minhas P., Parra L.C., Bikson M. (2012). Inter-Individual Variation during Transcranial Direct Current Stimulation and Normalization of Dose Using MRI-Derived Computational Models. Front. Psychiatry.

[84] Miranda P.C., Lomarev M., Hallett M. (2006). Modeling the current distribution during transcranial direct current stimulation. Clin. Neurophysiol..

[85] Workman C.D., Kamholz J., Rudroff T. (2020). Transcranial direct current stimulation (tDCS) for the treatment of a Multiple Sclerosis symptom cluster. Brain Stimul..

[86] Ho K.A., Taylor J.L., Chew T., Galvez V., Alonzo A., Bai S., Dokos S., Loo C.K. (2016). The Effect of Transcranial Direct Current Stimulation (tDCS) Electrode Size and Current Intensity on Motor Cortical Excitability: Evidence From Single and Repeated Sessions. Brain Stimul..

